# Children and Adolescents as Patients in General Practice - The Reasons for Encounter

**DOI:** 10.4021/jocmr597w

**Published:** 2011-07-26

**Authors:** Thomas Frese, Steffi Klauss, Kristin Herrmann, Hagen Sandholzer

**Affiliations:** a Department of Primary Care of the Leipzig Medical School, Philip-Rosenthal Strasse 55, 04103 Leipzig, Germany

## Abstract

**Background:**

The SESAM 2 study was performed to estimate consultations of general practitioners. In the recent work we focused on the reasons for encounter of children and adolescents consulting the general practitioner.

**Methods:**

Cross-sectional study with general practices in Saxony (Germany) as setting. Two hundred and seventy of the 2510 (10.8%) Saxon general practitioners agreed to participate and recorded data of 8877 patients. Evaluation of the data was organized by the Saxon Society of General Medicine (SGAM). Cross-sectional data were collected during a one-year period. One day of the week (Monday till Friday) was chosen at random for recording. Data were documented from every tenth patient with personal contact to the practitioner using a standardized report form at either the morning or afternoon consulting hours. Main outcome measures: reasons for encounter, the investigations and treatments performed and also the results of the consultation. Unpublished but publicly available data from the Dutch Transition Project were also analysed.

**Results:**

Eight hundred and five of 8877 patients were aged under 20 years. The mean percentage of children and adolescents in the general practice consultation was 9.1%. The mean number of reasons for encounter per child patient was about 1.5 and did not differ between the age groups. Most consultations were due to respiratory, digestive, skin or general symptoms with typical seasonal variations regarding the most frequent reasons for encounter caused by infectious diseases.

**Conclusions:**

As there is limited access to pediatric specialists, German general practitioners have to deal with children quite frequently. The number of child reasons for encounter is manageable for the general practitioner with an increasing spectrum of reasons for encounter among adolescents. In agreement with other publications most of the young patients consult for respiratory or general symptoms, or require preventive immunization or injection.

**Keywords:**

Children; Adolescents; Reason for encounter; General practice; Primary care

## Introduction

The traditional primary care disciplines of family practice, general internal medicine, and pediatrics provide comprehensive ambulatory care to broad population groups. In Germany primary care of children and adolescents is usually performed by general practitioners and pediatricians. General and family medicine per se can not exclude the treatment of children, defined as girls and boys under the age of 15 [1] and adolescents. However in children and adolescents, medical history, physical examination, differential diagnoses and treatment are different from those in adults. The percentage of children consulting general practitioners can be assumed to vary considerably. So treating these groups may be unusual for some general practitioners. Nevertheless, skills on a high professional level should be available for children and adolescents as patients in general practice, especially when there is limited access to pediatric specialists. General practitioners have to deal with acute and chronic diseases in children and adolescents. Acute disorders may induce chronic health problems in adulthood and may have a significant effect on the growth of the young patients [2]. Children are frequent visitors to general practitioners [3]. Though a high number of children suffer from long-term conditions like asthma, hay fever, allergy [4] or vision problems, the majority of the visits in primary care are for short term conditions or preventive reasons [1].

The general practitioner should keep in mind the most common reasons for encounter, the necessary procedures of taking history and physical examination as well as the most common diagnoses and their treatment regarding age-specific characteristics. However, the reasons of children and adolescents for consulting the general practitioner have not yet been systematically investigated. Overall, the percentage of papers on pediatric medicine in family medicine journals is small, although pediatric care is an essential part of family practice [5].

This investigation was set up to provide information on the reasons for encounter of children and adolescents. Therefore sub-group analyses of the SESAM 2 study [6] were performed to identify the most common reasons for encounters with focus on age-related differences and possible seasonal differences.

## Methods

The Saxon Society of General Medicine (SGAM) contacted all general practitioners in Saxony. Two hundred and seventy agreed to participate and 209 of the 2510 physicians cooperated. Cross-sectional data were collected from 1 October 1999 to 30 September 2000. Case recording was carried out on one day a week (Monday to Friday; either morning or afternoon consulting hours), chosen at random. Data were collected for one of ten patients previously known to the practitioner. Multiple screenings of the same patient were avoided. House calls were not considered. A total of 8877 patients were included. A standardized data collection form was used. It was developed by general practitioners (Leipzig Medical School and Saxon Society of General Medicine). The form was tested and evaluated during a pilot trial (SESAM 1). Each patients reasons for encounter, symptoms, diagnostic procedures, recent results of encounter / diagnoses and general morbidity were assessed as well as therapeutic procedures. As far as possible, data were documented verbatim (according to the study instructions), either as told by the patients (e.g. reasons for encounter) or in the physician’s words (e.g. chronic diagnoses). Due to the randomization pattern, the information was documented within a reasonably short time. Only completely filled-in forms were considered. The reasons for encounter were encoded following the ICPC [7, 8]. The SESAM 2 data were to be compared to those of other investigations. Unpublished, but publicly available data from the Transition Project (described by Okkes et al. [9]) were analyzed (total estimation; 1985 till 2003). The data are available at www.transitieproject.nl. They can be analyzed using the software that contains the database. For this, a comparable setting of data was chosen (1999-2000, single consultation without follow-up).

## Results

**Table 1:** Mean Number of Reasons for Encounter (RFE) per Patient, Patient Distribution (pd) on Different Age Groups in the SESAM 2 Study (as Childrens Rate of the Total Patient sample) Compared With Transition Project and With Frequency in German Population (FiP)

	SESAM	Transition Project	SESAM	SESAM	SESAM
Age (Years)	pd (%)	pd (%)	FiP* (%)	pd / FiP	RFE / patient
≤ 4	1.3	5.8	4.7	0.28	1.42
5 to 9	1.2	6.7	4.9	0.25	1.65
10 to 14	2.0	6.3	5.7	0.35	1.39
15 to 19	4.6	5.6	5.6	0.82	1.50
Sum	9.1	24.4	20.9	-	-

* data source: https://www-genesis.destatis.de/genesis/online (accessed march 23rd 2010).

**Table 2:** The 25 Most Common Reasons for Encounter (RFE) and Its Percentage in Different Age Groups (Data From the SESAM 2 Study)

ICPC	RFE	*N*_RFE_	% * in different age groups			
			0-4 (*n* = 112)	5-9 (*n* = 107)	10-14 (*n* = 179)	15-19 (*n* = 407)
R05	cough	112	10.69	17.05	6.83	7.84
A03	fever	102	10.06	11.93	8.84	7.03
A44	preventive immunization / injection	82	13.21	3.41	10.84	4.58
R21	throat symptom / complaint	73	2.52	5.11	6.83	7.03
R07	sneezing / nasal congestion	67	3.77	11.36	5.62	4.41
R31	respiratory: medical examination partial	38	7.55	3.41	4.42	1.47
A30	screening: medical examination complete	28	6.92	0.57	0.80	2.29
D01	abdominal pain / cramps general	27	0.63	1.14	2.01	3.10
D10	vomiting	27	1.89	3.98	2.41	1.80
D11	diarrhea	27	1.89	2.27	2.81	2.12
H01	ear pain / earache	20	0.63	3.41	1.20	1.63
N01	headache	20	0.63	-	0.80	2.78
R29	respiratory symptom / complaint other	20	3.14	2.27	1.61	1.14
D09	nausea	17	-	0.57	0.80	2.29
A04	weakness / tiredness	16	-	1.70	1.2	1.63
S56	dressing / compression / tamponade	16	1.26	1.70	2.01	0.98
A80	trauma / injury not other specified	14	-	-	0.40	2.12
H31	ear: medical examination partial	13	2.52	2.84	1.20	0.16
S06	rash localized	13	2.52	0.57	2.01	0.49
R22	tonsillar symptoms	12	-	-	1.61	1.31
S02	pruritus	9	1.26	1.70	0.80	0.33
D06	abdominal pain localized	6	-	0.57	1.20	0.33
S07	rash generalized	4	0.63	1.70	-	-
Sum of RFE		763	71.72	77.26	66.25	56.86

* percentage refers to all reasons for encounter per age group.

A total of 8877 consultations were recorded by 209 general practitioners. Eight hundred and five (approximately 9.1%) of these were contributed by patients from 0 to 19 years of age. The cases were reported by 172 general practitioners. The age distribution of these cases is summarized in Table 1. The different age groups had 1.3%, 1.2%, 2.0% and 4.6% of all estimated consultations. The mean number of consulting children and adolescents reported by each general practitioner was 4.68 (range 1 to 30, 1st quartile: 2, median 4, 3rd quartile: 6), while the mean number of all consultations reported by each general practitioner was 42.3 (range 23 to 54, 1st quartile: 39.5, median 43, 3rd quartile: 46). The percentage of children and adolescents in general practice ranged from 1.9% to 65.2% (1st quartile: 4.4%, median: 9.5%, 3rd quartile: 14.6%; mean: 11%). Based on all consultations, the seasonal percentage of children and adolescents consulting the general practitioner was between 10.2% (April) and 6.2% (August). Most encounters were initiated because of respiratory, digestive, skin or general symptoms with cough, fever, throat symptoms/complaints and sneezing/nasal congestion as the four most common non-procedural reasons for encounter (Table 2). It showed a clear seasonal distribution pattern (not shown). The comparison of the most frequent encounters between the SESAM 2 study and the Transition Project showed no remarkable differences (data not shown). This fact is also indicated by other studies [3, 4]. In the SESAM 2 study, the number of reasons for encounter per patient ranged from 1.39 to 1.65 between the age groups (Table 1). We found no sex-related differences in the reasons for encounter or its number per patient. The number of different reasons for encounter to manage a specific percentage of consultations is given in Table 3. Comparing data from the Transition Project to those of the SESAM 2 study revealed that there were more different reasons for encounter in the Transition Project. The number of regularly occurring reasons for encounter was about 15 to 26 in both studies (Table 4). This was somewhat lower than in adults and elderly (data not shown).

**Table 3:** Minimum Number of Different Reasons for Encounter (RFE) Needed to Manage a Given Percentage of Consultation in the Age Groups (Comparing SESAM 2 Study Versus Transition Project)

Age (years)	Study	Sum of all RFEs	*n*					
			50%	75%	90%	95%	97.5%	≈100%
0-4	SESAM	159	5	19	42	50	54	58
	TP	25 507	11	22	115	182	243	476
5-9	SESAM	176	5	16	39	48	53	57
	TP	19 953	16	29	140	210	283	502
10-14	SESAM	249	9	29	60	73	79	85
	TP	14 957	23	73	159	234	302	493
15-19	SESAM	612	14	44	90	121	137	151
	TP	21 333	33	95	201	283	366	630

**Table 4:** Number of Regularly (≥ 1 : 3000) Occurring Reasons for Encounter (roRFE) in Different Studies and Its Distribution by Age Group (Comparing SESAM 2 Study Versus Transition Project) Related to the Sum of All Encounters

	Age (years)							
	0-4		5-9		10-14		15-19	
Study	SESAM	TP	SESAM	TP	SESAM	TP	SESAM	TP
*n* (sum of all RFEs)	159	25 507	176	19 952	249	14 956	612	21 332
*n* (roRFE)	15	24	16	26	26	18	59	24

## Discussion

Our work is of scientific importance since it addresses a commonly discussed issue. Although pediatric care is an essential part of family practice, the percentage of papers about pediatric medicine in family medicine journals is small [5]. However, systematic investigations about the reasons for encounter of children and adolescents in a day-to-day setting have not yet been published. Existing studies either describe special reasons for encounter or the reasons for encounter of a particular age group [3, 4] or focus on the problems managed [1, 10]. The percentage of participating general practitioners was about 10%. However, regarding the results of other groups, the results can be assumed to be representative [11]. Because the SESAM 2 study not only focused on children and adolescents and estimated total morbidity, the assumption of attentional bias is unlikely. On the other hand a limit of the study is that training and experience in pediatrics of the participating practitioners and their attitude towards the treatment of children was not documented. Data sampling was conducted during a one-year period, thus seasonal bias was avoided. It should be kept in mind that after-hours calls and visits were not included. In this respect a small but highly necessary field of consultations still remains uninvestigated. The method of data sampling might have influenced the estimated results. However, data were collected from randomly selected patients, though the distribution amongst patients consulting general practitioners is not likely to be randomly. This study focuses on treatment of children in primary care not only in Germany but in the western world with similarities in health systems, so that its results can also be regarded as valid for the USA.

Bruijnzeels [12] reported the enormous amount of illness that occurs in children and the fact that more than 80% of all health problems are dealt with by parents without reference to the professional health care system. This was also stated by others [13]. More young children (0 - 4 years) suffered from illness generally and consultation rates differed widely according to symptoms [12]. The ratio of children and adolescents in the consultation and children and adolescents in the population support this (Table 1). The fact that there is no exclusive gatekeeper function of the general practitioner in the German health care system may also explain the low ratios from (patient distribution/frequency in population) of about 0.2. The present data indicate a high degree of variation in the percentage and number of children consulting each general practitioner. The average rate of about 9.1% is lower than those found in the Transition Project (approximately 24%) and reported by others (approximately 33%) [14]. In the group of 15 to 19-year-old adolescents the frequencies in consultation differ slightly (4.6% versus 5.6% comparing SESAM 2 to Transition Project; Table 1). These facts can be explained when regarding the parallel availability and free accessibility of pediatrics. In particular younger children may predominantly be seen primary by pediatricians. Forrest stated that the (self-) referral rate depends on the supply of specialists and the expectations for direct access to specialty care [15]. Also the treatment of small children may be feared by some general practitioners. The role of the German general practitioner as a gatekeeper and co-ordinator in the health care system turned out to be very limited [16]. It has become evident that a remarkably high number of general practitioners do not regularly treat children and adolescents in Germany. The variation in the number of children and adolescents consulting each GP might be explained by the different local availability of general pediatrics on the one hand and the attitude of the GP towards the treatment of especially young children on the other hand.

The number of reasons for encounter per patient (ranging from 1.39 to 1.65 between the age groups) is in accordance with the findings of other groups [17]. Reasons for encounter that occurred once per year or in 1 of 3000 cases in general practitioners’ consultation are qualified as usual [18]. Regarding the high variation of the percentage of children in each general practitioners’ consultation, defining usual reasons for encounter becomes difficult. This is because the rule of constant distribution of cases, as defined by Robert Braun, is not valid in this situation. As shown in Table 2, the percentage of the most common encounters based on all encounters decreased with age. While for younger patients reasons for encounter because of infectious diseases predominate [8, 13], adolescents consult general practice increasingly because of injuries, headache or contraception [3, 4, 19]. Therefore more different reasons for encounter have to be regarded to manage a specific percentage of all encounters in adolescents than in young children (Table 3).

Further research could focus on influences on management of child patients due to training and experiences in pediatrics, attitudes towards treating children or availability of general pediatrics.

### Conclusion

Children and adolescents are regularly seen by general practitioners in German primary care settings. Although there are differences in diagnostic and therapeutic procedures compared with adults, the treatment of children is familiar to general practitioners. The number of child reasons for encounter and therefore the spectrum of probable differential diagnoses is manageable for the general practitioner. There are about 25 regularly occurring reasons for encounter in general practice for children aged up to 19 years. The distribution of the most frequent reasons for encounter in the SESAM 2 study showed no remarkable differences in comparison to the Transition Project and other studies. Most of the young patients complained about cough, fever, throat symptoms and sneezing/nasal congestion but they also consulted for preventive immunization or injection. With increasing age the spectrum of reasons for encounter also increased.
